# JUAMI, the joint undertaking for an African materials institute: building materials science research collaborations and capabilities between continents

**DOI:** 10.1107/S2056989023010915

**Published:** 2024-01-26

**Authors:** Simon J. L. Billinge

**Affiliations:** a Department of Applied Physics and Applied Mathematics, Columbia University, New York, USA; IUCr, United Kingdom, and University of Bari, Italy

**Keywords:** collaboration, capability building, materials science, Africa

## Abstract

JUAMI, the joint undertaking for an African materials institute, is a project to build collaborations and materials research capabilities between PhD researchers in Africa, the United States, and the world. Focusing on research-active universities in the East African countries of Kenya, Ethiopia, Tanzania and Uganda, the effort has run a series of schools focused on materials for sustainable energy and materials for sustainable development. These bring together early-career researchers from Africa, the US, and beyond, for two weeks in a close-knit environment. The program includes lectures on cutting-edge research from internationally renowned speakers, highly interactive tutorial lectures on the science behind the research, also from internationally known researchers, and hands-on practicals and team-building exercises that culminate in group proposals from self-formed student teams. The schools have benefited more than 300 early-career students and led to proposals that have received funding and have led to research collaborations and educational non-profits. JUAMI continues and has an ongoing community of alumni who share resources and expertise, and is open to like-minded people who want to join and develop contacts and collaborations internationally.

## Abstract 2

JUAMI, the joint undertaking for an African materials institute, is a project to build collaborations and materials research capabilities between PhD researchers in Africa, the United States, and the world. Focusing on research-active universities in the East African countries of Kenya, Ethiopia, Tanzania and Uganda, the effort has run a series of schools focused on materials for sustainable energy and materials for sustainable development.

1.

It is a great milestone that the African Crystallographic Association (AfCA) has become a regional affiliate of the International Union of Crystallography and a testament to the growth of materials research in institutions on the African continent. It promises a bright future for materials research in Africa. In this article I describe a complementary effort with a goal of building international collaboration and research capabilities in materials science, specifically, to date, focusing on the east African countries of Ethiopia, Tanzania, Uganda and Kenya: the joint undertaking for an African materials institute (JUAMI). JUAMI is at its heart a community-building exercise, just as are professional associations such as AfCA. We would be very happy if, after reading this article, materials researchers will reach out and join the various JUAMI networking efforts and contribute to raising international collaborations in the community. More information is available at the JUAMI website (https://juami.org), and exchanges of questions, solutions and connections take place at the JUAMIexchange Google group (https://groups.google.com/g/juamiexchange) and the JUAMI LinkedIn page (https://www.linkedin.com/company/juami/). Please request to join either of these places if you would like to stay in touch.

The origins of JUAMI go back to 2010. Since the early 2000s the US National Science Foundation (NSF) Division of Materials Research (DMR) understood that materials research was taking place in sub-Saharan Africa, but apart from South Africa, there was a lack of institutions that could engage with NSF to build international efforts to enhance the activities. As part of their effort to increase the global awareness of the next generation of US researchers, and with a further goal of greater engagement in US–Africa cooperation, in 2010 the Division Director at the time, Zakya Kafafi, and Program manager Guebre X. Tessema reached out to three NSF-supported faculty, myself, Sossina Haile (then of California Institute of Technology, now Northwestern University) and Peter Green (then University of Michigan, now the National Renewable Energy Laboratory, a US Department of Energy laboratory) to join an NSF–DMR delegation to reach out to research-active institutions in East Africa to explore what new efforts could be of mutual interest. The JUAMI schools grew out of this. Discussions with research and teaching faculty, and university administrations, at Addis Ababa University (AAU), Makerere University (MU) in Kampala, the University of Nairobi (UN), the Nelson Mandela African Institution of Science and Technology (NM-AIST) in Arusha, Tanzania, and the University of Dar es Salaam indicated that capacity building and international collaboration focused on early-career scientists in the area of materials for sustainable development was something of great interest.

This resulted in a successful funding proposal to NSF from the US team of researchers to run a two week school at AAU on materials for sustainable energy that would bring together around 70 research-active PhD students from US universities and PhD, masters students and young faculty from universities in east Africa. In terms of the content, the aim was to deliver a mixture of (*a*) cutting-edge research talks from internationally renowned researchers, (*b*) interactive tutorial-level lectures on topics needed for research in this area but not part of basic curricula (such as the fundamentals of electrochemistry) and (*c*) hands-on team-building activities where students worked together in small groups (Fig. 1 ▸) to solve some experimental problem such as building an effective solar concentrator from cardboard and aluminium foil, or making a working dye-sensitized solar cell and measuring a voltage from the African sun. Beyond this, the goal was to encourage students to interact with each other, build greater understanding, collaborations and relationships that could be carried forward after the school was over.

The school was held at a hotel in Addis Ababa close to the main campus of AAU in December of 2012. The goal of cross-fertilization of ideas was taken seriously. Room-mates were assigned such that African and American students shared rooms; seating and group assignments were assigned randomly so people sat next to different people at each session and worked with different people in the groups. As well as the topical content, sessions focused on how to build collaborative proposals and even tips and tricks for effective communication. There were also group shared cultural activities.

The outcomes of the first school allowed successful proposals to be written for three more schools, in 2016 in Arusha, 2018 in Kampala and 2023 on the campus of UN. The basic goals and format of the schools has remained constant, though a number of aspects have been honed and modified. A strong effort has been made to maximize the diversity of opportunity with a target of 50% of women among each of the groups (US students, African Students and presenting faculty) and equal numbers of African and US students. These targets were reached in the last two schools. The original random sorting algorithm for seating and group assignments has been replaced with one that solves the maximally diverse grouping problem (MDGP), a pet project of the author. Peter Green has moved on to a senior role at DOE that doesn’t allow him time to continue with JUAMI, but Tom Mallouk (University of Pennsylvania) and Sara Skrabalak (Indiana University) have joined the US-side organizational team.

Finally, we have introduced a new outcome for the school, which is that students self-organize into groups during the second week and devise and create a funding proposal that proposes a solution to a problem identified by them in their discussions. This is presented by each group on the morning of the last day of the school. Originally, this was thought of as an exercise to teach effective collaboration and scientific communication, with feedback about how to make the proposal stronger and more likely to be supported. However, increasingly students have gone on to actually get the proposals funded and to develop the solutions. Some of these efforts have resulted in activities that persist over years and decades. As alumni of the schools transition from PhD student to post-doc to junior faculty, both in the US and in Africa, they bring their JUAMI experiences to their roles as teachers and researchers, for example, involving undergraduates at their institution in ongoing inter-continental research, education and outreach efforts.

One example is the SciBridge project (https://www.scibridge.org/). This grew directly out of one of the hands-on activities at the first JUAMI school, the construction and testing of the dye-sensitized solar cell. The palpable excitement when sunlight results in a measured voltage was compelling and the question was raised, could this activity be taken into undergraduate classrooms and even high schools in Africa to engage students in science and sustainability. The kits were not expensive to put together for US groups, but a challenge in African universities where funds for consumables are at a premium. The proposed solution was a partnership where kits were made in the US and shipped to African partners who did the outreach to local universities and schools. Classes were run by the African partners with remote or in person inputs from the US partners. This work continues today and has been expanded to more different hands-on activities.

Another outcome from the JUAMI schools was the development of a student-made potentiostat, a vital piece of laboratory equipment for electrochemistry (Fig. 2 ▸), but frustratingly expensive to purchase commercially. The project involved developing a low-cost potentiostat on an Arduino platform that could be put together for less than 100 USD compared to more than 1000 USD for a commercial potentiostat. The approach was published (Li *et al.*, 2018 ▸), has an online video showing how to put together your own potentiostat (https://www.juami.org/education/low-cost-potentiostat/). The paper has been cited more than 55 times (per Google Scholar), as well as multiple copies of the potentiostat being ‘manufactured’ and shared with partners within the JUAMI network.

Other projects that lived on beyond the school include creating educational modules in computational chemistry for use on iPads in Kenyan schools (Rodenbough & Manyilizu, 2019 ▸), the Women Supporting Women in the Sciences (WS2) effort to use materials science hands-on demonstrations and activities to promote retention of women in science in Africa and the US (https://ws2global.org/about-us), and the JUAMI open computing facility (JUAMI-OCF), which provides cloud computing to members of the JUAMI network. All of these, and others, were student-led initiatives with inter-continental cooperations that came about as a direct result of the JUAMI schools. As mentioned previously, a number of the initiatives were led by students who are now in permanent faculty positions and who continue them as part of their outreach efforts, giving new generations of students in their groups this global awareness and a taste of science as an international endeavour.

Success is hard to quantify in the world of international development, but JUAMI has had a lasting positive effect on many people, by their own admission (for example, see the videos at juami.org), and it continues to do so. Planning is beginning for the next phase of the JUAMI project and there is a strong desire to continue the activities in some form or another, possibly with a wider focus. There is a sense that materials research is picking up pace in sub-Saharan Africa and that efforts such as JUAMI, but also AfCA, the African Materials Research Society (AMRS), and other activities such as the African Light Source Initiative (AfLS to build X-ray light sources in Africa, https://www.africanlightsource.org/) can really help the research community in that region grow. Research funding agencies are starting to appear in these countries as well as institutions such as the African Academy of Sciences (AAS, https://aasciences.africa/). Such institutions in Europe and the US go back centuries in many cases, but higher education institutions in Africa are much younger. It is encouraging to see these institutions emerging and growing in Africa, not least AfCA, the topic of this special issue. The future looks bright and we encourage, especially young, scientists to engage in these efforts where they are able by joining and contributing to networks such as JUAMI, AMRS, AfCA and AfLS.

## Figures and Tables

**Figure 1:**
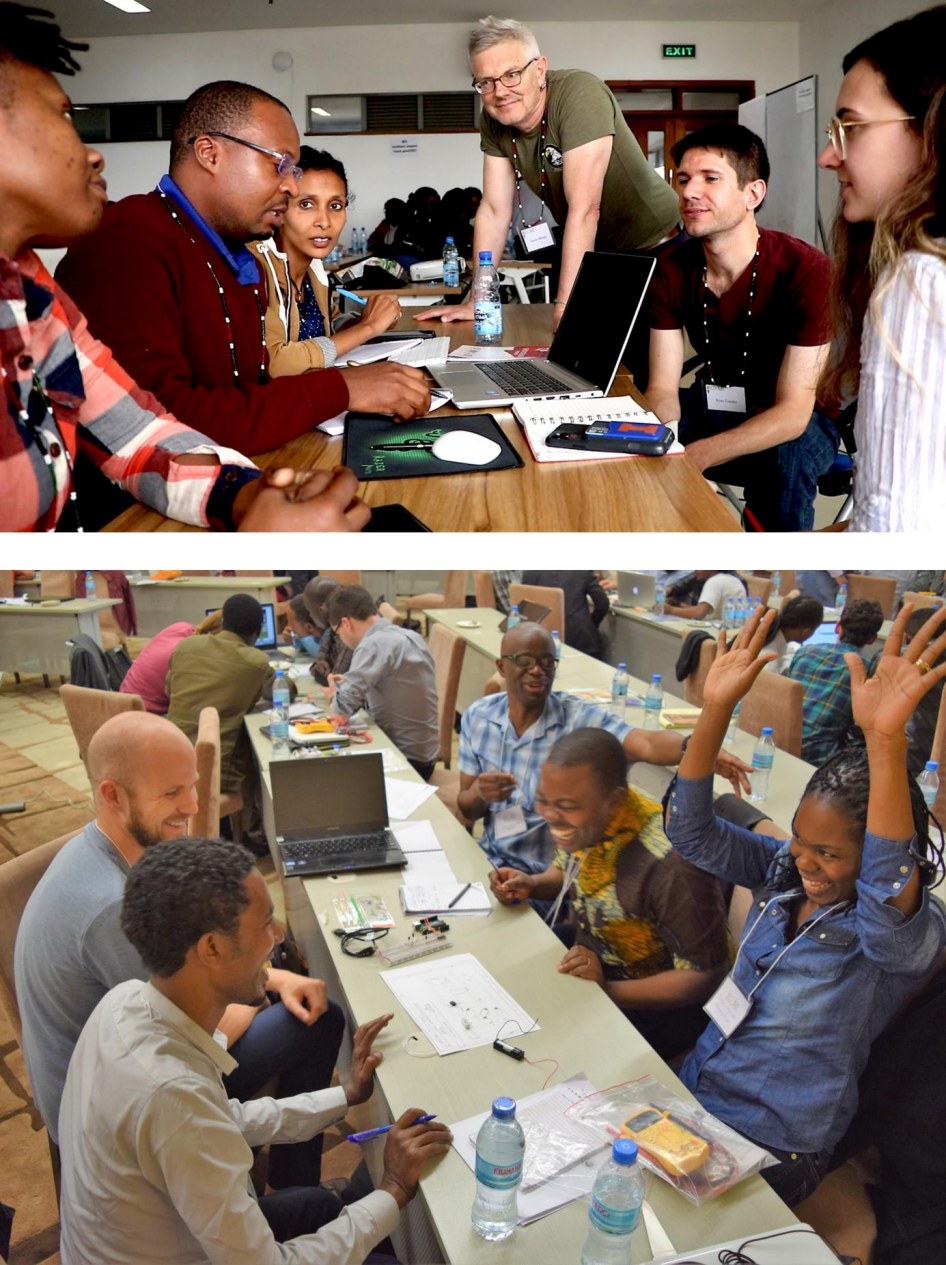
Top: The author and a group of JUAMI students at the 2023 school in Nairobi. Bottom: Students in a hands-on session at the 2016 ▸ JUAMI school in Arusha have some success.

**Figure 2:**
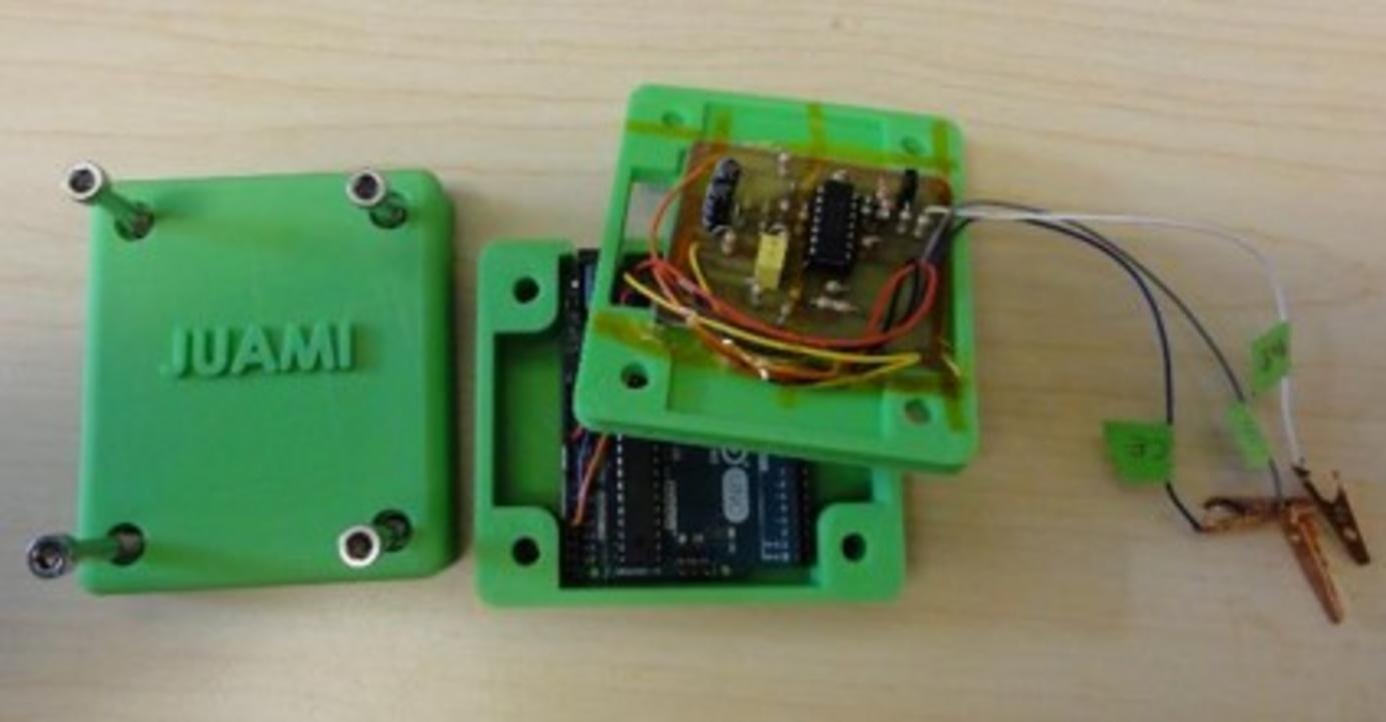
The JUAMI potentiostat, a student-led project to build a low-cost potentiostat for research and education.
